# Evaluation of a head rest prototype for rotational corrections in three degrees of freedom

**DOI:** 10.1002/acm2.14172

**Published:** 2023-10-04

**Authors:** Robert Schindhelm, Gary Razinskas, Jonas Ringholz, Johannes Kraft, Otto A. Sauer, Sonja Wegener

**Affiliations:** ^1^ Radiation Oncology University Hospital Wurzburg Wurzburg Germany

**Keywords:** cranial irradiations, patient positioning, rotational corrections

## Abstract

Cranial stereotactic irradiations require accurate reproduction of the planning CT patient position at the time of treatment, including removal of rotational offsets. A device prototype was evaluated for potential clinical use to correct rotational positional offsets in image‐guided radiotherapy workflow. Analysis was carried out with a prototype device “RPS head” by gKteso GmbH, rotatable up to 4° in three dimensions by hand wheels. A software tool accounts for the nonrectangular rotation axes and also indicates translational motions to be performed with the standard couch to correct the initial offset and translational shifts introduced by the rotational motion. The accuracy of angular corrections and positioning of an Alderson RANDO head phantom using the prototype device was evaluated for nine treatment plans for cranial targets. Corrections were obtained from cone beam computed tomography (CBCT) imaging. The phantom position was adjusted and the final position was then verified by another CBCT. The long‐term stability of the prototype device was evaluated. Attenuation by the device along its three main axes was assessed. A planning study was performed to evaluate if regions of high‐density material can be avoided during plan generation. The device enabled the accurate correction of rotational offsets in a clinical setup with a mean residual angular difference of (0.0 ± 0.1)° and a maximum deviation of 0.2°. Translational offsets were less than 1 mm. The device was stable over a period of 20 min, not changing the head support plate position by more than (0.7 ± 0.6) mm. The device contains high‐density material in the adjustment mechanism and slightly higher density in the support structures. These can be avoided during planning generation maintaining comparable plan quality. The head positioning device can be used to correct rotational offsets in a clinical setting.

## 1 INTRODUCTION

Stereotactic radiotherapy is a commonly used treatment technique for brain metastases.^1^ Quality requirements exist for such a technique enabling highly precise and accurate dose delivery.^2^ Typically, high doses are applied with a high degree of conformity in one or a few fractions following image‐guided patient positioning.

When individual targets are relatively small and spherical and the isocenter is located in the center of the target, translational setup corrections were reported as sufficient.^3^ However, rotational setup errors result in reduced target coverage with increasing isocenter‐to‐target distance.^4^, ^5^ In recent years, the simultaneous irradiation of multiple brain metastases using a single isocenter has become more common.^6^, ^7^ Failure to correct rotational setup errors can result in PTV underdosage.^8^ This can be mitigated by adding an increased margin when creating the planning target volume (PTV), but is then associated with an increased dose to healthy brain tissue and could potentially pose a higher risk of adverse radiation effects.^9^

It is still debated whether rotational corrections are necessary for a wider range of extracranial anatomical sites. Typically, rotational errors have little impact on target doses when appropriate margins are used for the creation of the PTV, such as 5 mm for head and neck.^10^ However, the effect on individual fractions or systematic impacts on organs at risks can be more pronounced.^10^, ^11^ Reduced target coverage is of clinical relevance especially for elongated targets close to organs at risk.^12^ For those reasons, the use of six degrees of freedom (6DOF) couches is typically recommended.^10^, ^11^, ^12^, ^13^

In addition to the dosimetric improvements, rotational corrections facilitate the patient setup workflow. For head and neck patients, 2.4 min per treatment could be spared, less patient repositioning and fewer cone beam CTs were required when working with a 6DOF instead of a 4DOF couch (translational motions and yaw rotation).^14^

Corrections in 6DOF are typically performed with robotic couches.^15^, ^16^, ^17^ However, 6DOF couches are not available on all treatment machines and are associated with extra costs.^18^ A planning study on whole brain irradiation with hippocampal avoidance and simultaneously integrated boost volumes for the increasingly common Varian Halcyon mentions the inability to perform corrections of rotational setup errors as one of the main limitations for the clinical use of this technique.^19^

A head immobilization device that allows the rotational adjustment of patient positioning provides an alternative solution to the use of a 6DOF couch. So far, a head‐supporting device was presented, which is restricted to corrections of pitch and yaw.^20^ The RPS head by gKteso GmbH (Bobingen, Germany) is a new device, allowing rotational corrections of the patient head around the three axes pitch, roll, and yaw. Translational motions can be performed with the standard couch. Two workflows are suggested for the device. First, positioning instructions can be obtained directly from a surface‐scanner if the treatment machine is equipped with a system for surface‐guided radiotherapy (SGRT) that displays rotational and translational offsets. Alternatively, rotational and translational offsets can be obtained from CBCT images if image‐guided radiotherapy (IGRT) is used for positioning. These offsets are further processed with a software to yield positioning instructions.

In this study, the newly introduced RPS head was evaluated for clinical use using the IGRT workflow. This included the analysis of the accuracy of the corrections of the device itself and during the standard workflow. In further tests, its stability and attenuation were considered.

## 2 MATERIALS AND METHODS

**FIGURE 1:**
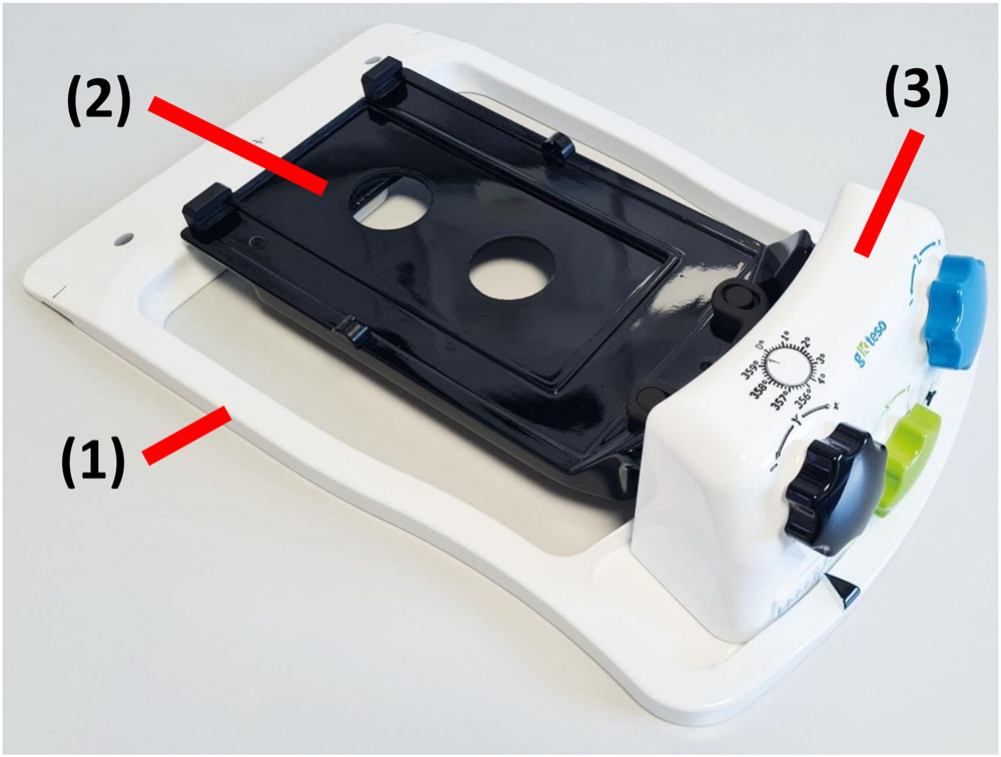
RPS head prototype for rotational corrections. The base frame (1) can be fixed on the treatment couch by an index bar. The patient is fixated on the rotational head support plate (2) using a head support and a thermoplastic mask. The rotation mechanism is included in the housing (3) and is operated manually by adjusting the hand wheels.

The head fixation device “RPS head” consists of the following main components (Figure 1): (1) A base frame that can be mounted on the treatment couch using an indexing bar. (2) A rotatable head support plate fixed to the base frame by a ball joint assembled near the neck side. For positioning the patient on this plate, a head rest as well as a thermoplastic mask can be utilized. (3) An adjustment mechanism is located at the cranial end of the base frame. With three integrated hand wheels—that is, one per rotation axis and a 0.2° interval scale each—the rotation of a patient's head can be adjusted. Depending on the rotation axis, the device is capable of rotations up to ±3° (yaw) or ±4° (pitch and roll). In the following, the head of an anthropomorphic Alderson RANDO phantom was used for analyzing the achievable accuracy of angular corrections. The measurements were carried out using an Elekta Synergy Linac (Elekta Oncology Systems, Crawley, UK) including cone beam computed tomography (CBCT) and its XVI software (version 5.0.4). The phantom with the RPS head device was moved with either the 6D robotic Hexapod couch and the iGuide software (Medical Intelligence, Schwabmuenchen, version 2.2.3) or the standard Elekta Precise couch.

### 2.1 Accuracy of adjusted rotation angles

For testing the accuracy of rotational adjustments, a total of 17 rotational errors from 0° to ± 3° in a single of the three rotational directions were introduced by a 6D robotic couch. The RPS head was used with the head of an Alderson RANDO phantom attached to the rotatable head support plate with a tightly fitting thermoplastic mask. The measurement procedure applied here was based on the following repetitive steps:

- Setting the RPS head hand wheels to zero rotation. Rotating the RPS head as a whole around a single rotational axis using the 6D robotic couch to generate the intended rotational error.
- Performing a CBCT to quantify the introduced error by matching the image against a reference in the XVI software. Correcting the error by rotating the RPS Head according to the XVI measurement.
- Performing a CBCT to verify the resulting position and remaining rotational offset of the device.

For comparability, all CBCTs were performed in the same rotational direction using a head preset and a full rotation. Furthermore, CBCT and planning CT were automatically aligned using the XVI software and including the bones of the whole cranium into the match. Automatic matching was reviewed to spot any gross errors.

### 2.2 Full end‐to‐end test including the correction function

When correcting translational and rotational displacements with the standard couch in conjunction with the RPS head device, two device properties have to be considered due to its construction. First, the roll axis is not perpendicular to the pitch and yaw axes but stands at an angle of about 8° to the base plate. Second, because of the distance between the ball joint and the target point, rotations of the device are accompanied by translational shifts of the patient's head—for example, a rotation around the pitch axis causes an up or down movement. Corrections for these movements are necessary when adjusting the device to reproduce the head position of the planning CT. Therefore, a correction function to account for additional translations as a function of the rotation angle and the relative coordinates of the target point with respect to the ball joint is implemented in the vendor‐provided software. The software automatically obtains all necessary information, namely current couch positions and intended offsets, from its XVI interface and provides the user with recalculated angular corrections to be applied using the hand wheels and target couch coordinates for the translational shifts.

The full clinical workflow was validated. In total, nine treatment plans were generated with target points located in different areas of the brain region of the Alderson RANDO head phantom. After the RPS head device is fixed to the couch, the couch has to be adjusted in order to match markers on the RPS head with the room lasers. In this way, the couch coordinates of the RPS head base plate are captured by the software. Together with the coordinates in final treatment position, the correction function is able to execute the position depending corrections.

Small errors were introduced by changing the couch position and by rotating the Alderson RANDO phantom. For this purpose, a small spacer was placed in different positions between the rotatable head support plate and the head rest. Afterwards, a CBCT was performed to quantify the magnitude of the introduced offsets and acquire the necessary corrections from the XVI software.

The obtained XVI correction values served as input parameters for the correction function to recalculate the rotational and translational adjustments. The software obtains the values automatically from an XVI interface and displays the final necessary corrections. The translational shifts were applied manually inside the treatment room with the Elekta Precise couch until final positions were reached. The given corrected rotational values from the vendor's software were applied manually to the RPS head. An additional CBCT was performed to obtain residual setup errors after the correction procedure.

### 2.3 Long‐term stability of the device

The stability of the RPS head was investigated over 20 min in 2‐min intervals to mimic the time for a stereotactic treatment. For this test, a volunteer was positioned on the RPS head with the standard immobilization tools used for cranial stereotactic radiotherapy, which are a head rest and an individually fitted thermoplastic mask. Measurements started right after positioning the volunteer on the couch. A laser distance‐measuring device (Leica DISTO D210, Leica Geosystems, Heerbrugg, Switzerland) was fixed about 30 cm above the couch and spotted to a marker on the rotatable plate. The distance between the distance‐measuring device and the marker was determined. The marker was located close to the caudal end of the rotatable head support plate and off the symmetry axis. When adding additional force to the plate, this point was rather flexible, especially with regard to roll and pitch movements. The location off‐axis implies that translational motion as well as both roll and pitch movements affect the distance between the marker and measuring device.

Data were collected for three different setup scenarios. First, stability was measured without rotating the device. Next, all axes of the RPS head were rotated by +2° and then by −3°. In this way, sagging of the device in three extreme positions could be examined. Between each measurement, the device was unloaded for 10 min. Upon completion of data collection, the volunteer was asked to strongly move the head left, right, up, and down to further analyze the stability of the RPS head.

### 2.4 Attenuation measurements

To identify regions with high attenuation in the RPS Head device, portal images were acquired with a Halcyon linear accelerator (Varian Medical Systems, Palo Alto, CA) since these images could be dosimetrically evaluated in the Portal Dosimetry application of ARIA (V15.6, Varian Medical Systems). To bypass the inhomogeneous beam profile of the linac, dynamically flattened beams were irradiated while the device was positioned in the beam path. In order to measure all three main planes, three different configurations were utilized: (i) RPS head horizontally on the couch, and gantry angle 0°, (ii) RPS head unchanged and gantry angle 270°, (iii) RPS head vertically on the couch, and gantry angle 0°.

For the three structures within the RPS head, the attenuation was determined by point measurements. For this purpose, a Semiflex 31013 ion chamber connected to a Unidos electrometer (both PTW‐Freiburg, Freiburg, Germany) in a 10 × 10 × 6‐cm^3^ PMMA block was positioned on the RPS head in the isocenter of an Elekta Synergy Agility linac. A 10 × 10‐cm^2^ 6MV field in one of the three setups was realized: (i) gantry at approximately 130° such that the ion chamber was behind a bar of the base plate in the beam's eye view, (ii) gantry at 180° with the rotational head support plate in the beam path, and (iii) gantry close to 90° and couch close to 270° such that the beam passed through the operating housing directly through one of the hand wheels before reaching the ion chamber. The exact geometry was determined with the aid of the light field. The ion chamber signal for 100 MU for each configuration was obtained with the RPS head present and then again after removing the RPS head and repositioning the PMMA block in the isocenter guided by the lasers using a polystyrene block providing the same height as the RPS head. The ratio between the signal with and without RPS head present was calculated and is referred to as attenuation.

### 2.5 Planning study

The feasibility of irradiations with the RPS head was evaluated from the treatment planning perspective using the Pinnacle system (Philips Radiation Oncology Systems, Fitchburg, WI, version 16.2). In a first step, the attenuation due to the inclusion of the RPS head device was assessed. The device density was removed from a planning CT image of an Alderson RANDO head phantom. In more detail, the volume above the cranium, which is in particular the adjustment mechanism as well as its operating housing, was overridden with air. VMAT plans were generated for seven targets evenly distributed within the brain region. Each plan was composed of one noncoplanar half rotation arc with the couch at 270° and one or two planar full‐rotation arcs. Afterwards, a copy of the plan was generated on a CT containing the device density information. Since the exact composition of the RPS head was not revealed by the manufacturer, high‐density elements of the adjustment mechanism were overridden with aluminum density. This was based on the density values displayed in the planning CT of approximately 2950 HU acquired with a SOMATOM go.Open Pro (SIEMENS Healthineers) using a calibrated HU to density curve capable of accurately representing HU values up to a maximum of 65 536 HU (16 bit). The prior generated VMAT plans were recalculated on this alternative density CT data keeping the field details and monitor units unchanged. Differences between the two plans in terms of target volume dose–volume histogram parameters D99, D98, D95, D2, and D1%, and V99, V95, and V90% and maximum dose to the outer 5 mm of the body contour were investigated.

Second, the feasibility of creating treatment plans including the RPS head was evaluated. The VMAT plans were re‐optimized with the same setup of gantry and couch on a CT image including the RPS head density with high‐density material component overrides. Dose–volume parameters and the number of monitor units of the respective arcs were obtained and compared to the plans optimized on the CT with all RPS head density removed. Additionally, the effect of small positional changes of the RPS head on the dose calculation was investigated. Relative displacement between the RPS head high‐density components and the target region could arise due to interfractional setup variation or the adjustment of the RPS head device. Relative offsets were mimicked by shifting the RPS head density elements in the CT by 6 mm in lateral and 8 mm in longitudinal and vertical direction, which are typical translational displacements due to the correction of setup rotations of up to 3° with the RPS head. Target dose parameters were obtained for the recalculated plans keeping the same beam geometry and MU and compared to the calculation with the RPS head in its initial position.

## 3 RESULTS

### 3.1 Accuracy of adjusted rotational angles

Rotational errors could be corrected to (0.0 ± 0.1)° accuracy around each of the three rotational axes (Table 1). The maximum residual rotational offset after correction was 0.2°.

**TABLE 1:** Residual rotational offsets after rotational corrections around the specified axis using the RPS head.

	Pitch	Roll	Yaw
Mean (°)	0.0	0.0	0.0
1 SD (°)	0.1	0.1	0.1
Maximum (°)	0.2	0.2	0.2

### 3.2 Full end‐to‐end test including correction function

Setup corrections in 6DOF using the RPS head in conjunction with the corresponding software applying the correction functions allowed reliable repositioning (Table 2). Residual rotational setup errors were accurate to (0.0 ± 0.1)°, (0.0 ± 0.2)°, and (0.0 ± 0.1)° around the pitch, roll, and yaw rotational axes. The maximum residual rotational offsets around the respective axes were 0.1°, 0.4°, and 0.2°. Translational errors could be corrected with the following residua: Δ*x* = (−0.2 ± 0.5) mm, Δ*y* = (−0.2 ± 0.5) mm, and

**TABLE 2:** Residual translational and rotational offsets after setup corrections following the full clinical workflow using the RPS head for nine treatment plans with different isocenter positions.

Target	Setup error before correction						Residual setup error after correction					
	*x*/mm	*y*/mm	*z*/mm	Pitch/°	Roll/°	Yaw/°	*x*/mm	*y*/mm	*z*/mm	Pitch/°	Roll/°	Yaw/°
1	−2.1	1.5	2.2	1.7	2.2	−0.1	0.6	−0.1	0.6	0.0	0.1	0.0
2	−1.4	−2.2	1.2	13	2.7	−1.2	−0.9	0.0	0.0	0.0	−0.1	−0.1
3	−3.8	4.7	4.0	1.5	2.6	−2.1	0.3	−1.0	0.6	−0.1	−0.1	−0.2
4	−5.8	−5.4	4.4	−2.8	−2.6	−0.3	−0.1	−0.4	0.5	0.0	0.4	0.0
5	15.3	−2.7	4.6	−1.3	2.7	2.2	−1.0	0.6	−0.6	0.1	−0.4	0.0
6	−8.7	1.4	2.3	0.2	2.5	−0.4	−0.4	0.2	−0.6	0.1	0.1	0.1
7	−2.2	−6.2	0.2	0.2	1.5	0.4	−0.1	−0.3	−0.1	0.0	0.0	0.0
8	−0.2	−6.4	−2.8	0.7	2.9	0.0	−0.1	−0.7	−0.1	0.0	0.0	0.0
9	6.9	−2.6	1.6	−0.9	2.5	−0.1	−0.3	0.1	0.0	0.0	−0.2	0.0
**Mean**							**−0.2**	**−0.2**	**0.0**	**0.0**	**0.0**	**0.0**
**1 SD**							**0.5**	**0.5**	**0.5**	**0.1**	**0.2**	**0.1**
**Max. residuum**							**−1.0**	**−1.0**	**±0.6**	**±0.1**	**±0.4**	**−0.2**

Δ*z* = (0.0 ± 0.5) mm. The stated uncertainty is one standard deviation.

### 3.3 Long‐term stability

**FIGURE 2:**
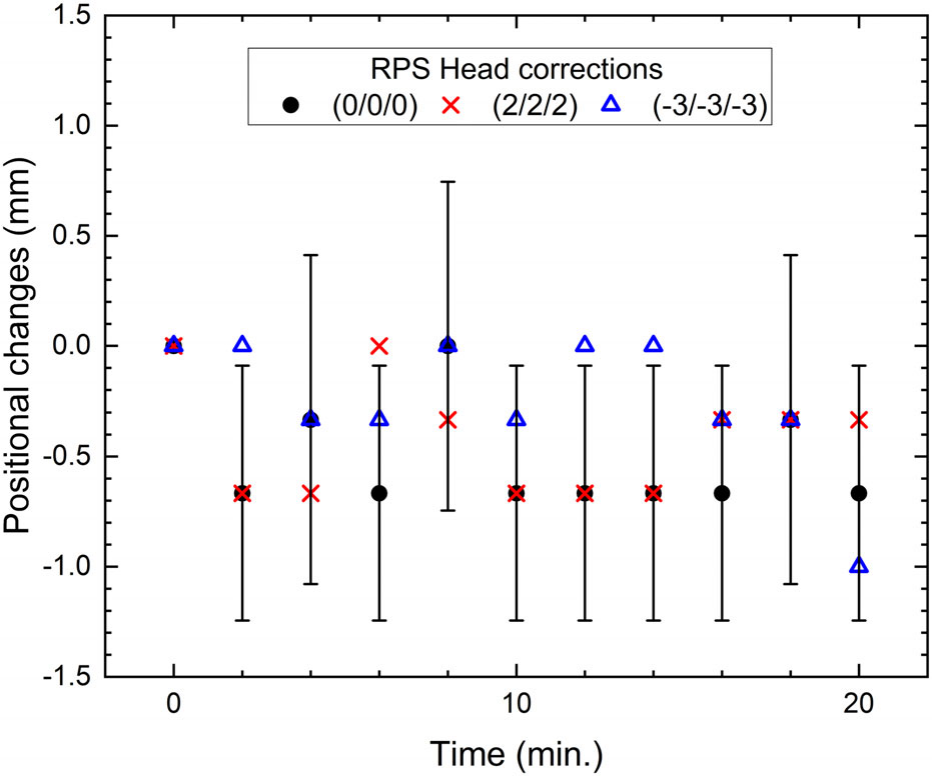
Positional changes of the RPS head support plate versus a fixed point as a function of time. A volunteer was positioned on the device and angular corrections (pitch/roll/yaw) were applied at the time *t* = 0. The numbers in brackets indicate the rotational setting around each of the rotation axes. Negative values indicate a sagging in direction of gravitation.

For the initial setup without any rotation of the RPS head, the measured distance between a fixed point and the RPS head support plate was stable within (0.7 ± 0.6) mm over the 20‐min observation period (Figure 2). The uncertainty includes contributions from three repeated measurements (one standard deviation) and the measurement accuracy of the laser measuring device. Fluctuations for cases with an initial large angular adjustment of the device yielded comparable shifts. Purposely introduced strong head movements of the volunteer introduced positional changes no greater than 2 mm. It should be noted that such an exertion of force against the positioning device should not be the clinical norm.

### 3.4 Attenuation

**FIGURE 3:**
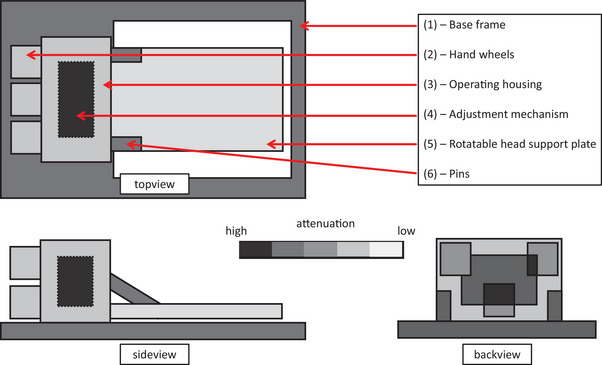
Schematic map of the device's attenuation of the 6‐MV beam as recorded on the portal imager from three directions.

Portal images indicate the distribution of high‐density material within the positioning device (Figure 3). Highly attenuating materials are found especially in the adjustment mechanism and in the supports. Point measurements yield an attenuation of 26.5% for the beam passing through the adjustment mechanism and 10.0% for the beam passing through the side bar of the base frame, with 1.2% as one standard deviation. Attenuation by the rotational head support plate is small (0.8%).

### 3.5 Planning study

The insertion of the RPS head into the treatment plans affected the calculated dose distribution. On average, the dosimetric quantities D99, D98, and D95% of the target volumes decreased by (4.3 ± 1.5), (4.2 ± 1.5), and (4.2 ± 1.5)%, respectively. D2 and D1% decreased by (3.1 ± 1.5) and (3.1 ± 1.5)%. V99 and V95% decreased by (78 ± 32) and (5.7 ± 9.4)%, respectively. V90% yielded no difference. There were no relevant differences between the dose distributions of the original plans without the RPS head density and those obtained from re‐optimizing plans after inserting and considering the positioning device density including its high‐density elements. The largest differences were observed for D99 and D2% with an average deviation of (0.1 ± 0.2)%. The maximum skin surface dose within 5 mm of the body contour increased by (0.7 ± 1.8)% of the prescribed target dose for the re‐optimized plans.

In the re‐optimized plans, the total number of MU increased slightly by (6.1 ± 5.1)% in favor of the coplanar arcs. The distribution of the MU between the arcs remained almost unchanged after re‐optimization. The fraction of MU allocated to the co‐planar arc was reduced by (2.2 ± 4.4)% on average.

Small displacements of the positioning device relative to the head phantom did not noticeably affect target coverage. For lateral, longitudinal and vertical shifts D99% decreased by an average of (0.16 ± 0.11), (0.09 ± 0.10), and (0.16 ± 0.11)%, respectively.

## 4 DISCUSSION

The head immobilization device RPS head enabled a full correction of rotational offsets with a maximum residual error of 0.2° despite manual adjustment according to the reading of the angular scale and the repeatability of the XVI match. Furthermore, the integrity of the provided correction function in the clinical workflow as well as the interface to XVI could be tested successfully. Positional misalignments of the head phantom in translational and rotational direction could be corrected. Rotational offsets were corrected to 0.0° with a standard deviation of 0.1° or 0.2° for all rotational axes following the entire clinical IGRT workflow. In two cases, a maximum residual error of 0.4° remained around the roll axis. The deviations can mainly be attributed to mechanical play of the head phantom within the immobilization mask, repeatability of the XVI match, and manual adjustment of several axes at a time. No CBCT image artifacts were visible in the CBCT that interfered with matching. The high‐density material in the adjustment mechanism is located roughly 10 cm above the cranium.

Translational shifts due to the slightly nonorthogonal rotational axes and the distance between ball joint and target point could be counteracted with residuals below 1 mm. The RPS head provides accurate rotational correction comparable to other mechanical and robotic positioning devices. The couch‐top correction device characterized by Usui et al.,^20^ which only enables yaw and pitch corrections, was accurate within 0.5°. The robotic Hexapod couch in combination with the XVI system was also characterized as accurate within 0.3°.^21^ Translational offsets are better corrected with the robotic couch to below 0.3 mm^21^ compared to the maximum of 1.0 mm in the clinical workflow in combination with the Precise couch motion. The translational accuracy of the RPS head following the entire clinical setup workflow not only depends on the device itself and its correction function but is also limited by the correct XVI match, head immobilization, and the movement accuracy of the Precise couch. The precision of the calibration of the couch movement depends on the individual couch and direction of movement. Riis and Zimmermann^22^ reported deviations of up to 9.1 mm/m for the vertical axis on one of their machines and oscillations of about 1 mm when using automatic remote positioning (ASU). The major contribution to the setup uncertainty in our case is the couch coordinate being displayed with 1‐mm resolution by default. Overall, the accuracy of the entire workflow with the phantom is comparable to what is achievable with fixated patients, where Guckenberger et al.^16^ reported <1‐mm translational and <1° rotational accuracy. The maximum displacement observed here was also within the 1.3‐mm displacement expected from interfractional motion on a rigid device, as detailed by Engelsman et al.^23^

The stability of the device over time could be verified with the help of a volunteer. The use of the volunteer instead of the head phantom includes additional weight or weight shifts by the neck and shoulders. Sagging of the device was at maximum (0.7 ± 0.6) mm observed over a period of 20 min after performing three different rotational corrections. The associated uncertainties result mainly from the uncertainty of the laser measurement device. The 20‐min interval should cover the realistic time for a stereotactic treatment. It is recommended that the time between imaging and the start of treatment is no more than 5 min.^24^ Exemplary treatment times for stereotactic brain metastases on a LINAC were reported as 5.6–9.1 min depending on the number of targets.^25^ Both sum up to approximately 10–15 min. Therefore, 20 min should not usually be exceeded. This confirmed the robustness of the RPS head and its suitability for long stereotactic treatments. The long‐term stability should be monitored for a longer period where longer treatment times are common. Nevertheless, should the cumulative patient setup and treatment duration surpass 20 min, it is advisable to consider a re‐imaging process, owing to potential patient movements.

Since the rotational corrections have to be applied manually by turning the hand wheels on the RPS head, a second CBCT after manual adjustments is inevitable. Although imaging is a small contribution to patient dose, the acquisition of two CBCTs per fraction during stereotactic brain irradiation is not unusual.^3^, ^26^ The differently colored hand wheels and angular scales on the RPS Head facilitate the adjustment process for the user. Although fully functional in its current form, automated communication between correction function, XVI and couch as well as an automated adjustment of the positioning device is a desirable alternative in the future.

The attenuation of treatment beams by the RPS head varied depending on the target position in the head phantom and the chosen gantry and couch angles. When the density and the structure of high‐density elements are considered accurately before optimization, target coverage is not compromised. The generated plans were robust against small shifts of the head relative to the positioning device. Whenever possible, the planner should avoid entry angles that penetrate or are close to the mechanics of the RPS head or avoid the high‐density regions during optimization. Although attenuation and scatter of the beams by the mechanical elements may be accounted for by treatment planning, the position of high‐density elements may change relative to the patient anatomy. Therefore, treatment planning should avoid beam entry angles that penetrate the mechanics of the RPS head in the planned position and in all geometries resulting from positional corrections. For inverse optimization, two separate arcs excluding the unfavorable angular range can be used or a respectively low maximum dose value constraint for the high‐density region can be added to reduce contributions from incident beams penetrating or close to the high‐density material. Since we analyzed the attenuation on a prototype, we recommend confirming these attenuation properties for the final product.

The RPS head allows accurate positioning following the clinical workflow. To ensure a reproducible position of the patient head from planning CT to treatment, the head immobilization device has to be used in the planning CT as well. Alternatively, a device with the same dimensions as the RPS head but without the mechanics for rotations may be utilized.

## 5 CONCLUSION

The RPS head positioning device in conjunction with the software to calculate rotational and translational corrections can be used to correct rotational offsets in a clinical setting for IGRT treatments with high accuracy. Potential areas of application are treatment machines that were purchased without a robotic couch or for which no robotic couch is offered. If the high‐density parts of the positioning device are taken into account during treatment plan generation, there is little dosimetric effect on target coverage or skin dose.

## AUTHOR CONTRIBUTIONS

Robert Schindhelm, Otto A. Sauer, and Sonja Wegener designed the study. Robert Schindhelm, Sonja Wegener, Gary Razinskas, and Jonas Ringholz collected the data. All authors discussed the results. Robert Schindhelm and Sonja Wegener wrote the manuscript in consultation with all authors. All authors agree with the publication.

## CONFLICT OF INTEREST STATEMENT

RS, OS, and SW received travel grants from gKteso to present preliminary results at international conferences. There are no other potential conflicts of interest to disclose.
